# Microvascular capillaroscopic abnormalities and occurrence of antinuclear autoantibodies in patients with sarcoidosis

**DOI:** 10.1007/s00296-022-05190-5

**Published:** 2022-08-30

**Authors:** Francesco Cattelan, Elvis Hysa, Emanuele Gotelli, Carmen Pizzorni, Pietro Francesco Bica, Marco Grosso, Emanuela Barisione, Sabrina Paolino, Luca Carmisciano, Alberto Sulli, Vanessa Smith, Maurizio Cutolo

**Affiliations:** 1 Laboratory of Experimental Rheumatology and Academic Division of Clinical Rheumatology, Department of Internal Medicine, University of Genoa, IRCSS San Martino Polyclinic, Viale Benedetto XV, 6, 16132 Genoa, Italy; 2 Pneumology Unit, IRCCS San Martino Polyclinic, Genoa, Italy; 3 Department of Health Sciences, University of Genova, Genoa, Italy; 4 Unit for Molecular Immunology and Inflammation, VIB Inflammation Research Center (IRC), Ghent, Belgium; 5 Department of Internal Medicine, Ghent University, Ghent, Belgium; 6 Department of Rheumatology, Ghent University Hospital, Ghent, Belgium

**Keywords:** Sarcoidosis, Microscopic angioscopy, Antinuclear antibodies, Microcirculation

## Abstract

We described nailfold videocapillaroscopy (NVC) findings and estimated the prevalence of serum anti-nuclear (ANA) and extractable nuclear antigen autoantibodies (ENA) in a cohort of sarcoidosis patients, comparing them with adequate healthy controls (HCs) and with primary Raynaud’s phenomenon patients (PRPs). NVC findings were also correlated with the occurrence of autoantibodies, current treatment, laboratory parameters, variables of lung function and whole-body imaging data. Twenty-six patients with sarcoidosis were assessed through NVC, laboratory parameters, pulmonary function tests, chest-X ray and 18- fluorodeoxyglucose positron emission tomography/computed tomography. The NVC parameters and ANA/ENA dosage were recorded also in 30 PRPs and 30 HCs. Sarcoidosis patients showed a higher rate of capillary dilations and nonspecific abnormalities and a lower mean capillary absolute number than PRPs and HCs (*p* < 0.01 for all comparisons). The prevalence of ANA positivity was higher in patients with sarcoidosis compared with PRPs and HCs (*p* < 0.02 for both), whereas ENA positivity was detected in one sarcoidosis patient (Ro52). Among sarcoidosis patients, the mean capillary absolute number negatively correlated with the C-reactive protein concentrations and was positively associated with the forced vital capacity percentage. Instead, a negative correlation was detected between serum ACE levels and the presence of capillary dilations (all *p* < 0.05). Our findings suggest a microvascular involvement in sarcoidosis whose investigation by NVC might be useful for the follow-up of patients displaying RP. Autoantibody positivity in sarcoidosis might suggest autoimmune implications in the disease or the production of autoantibodies reactive to tissue damage.

**Supplementary Information:**

The online version contains supplementary material available at 10.1007/s00296-022-05190-5.

## Introduction

Sarcoidosis is a systemic granulomatous disease with multi-organ involvement [1–3]. Its pathogenesis is complex: in a genetic background, multiple environmental factors elicit an innate and an adaptive immune response with the hyper-activation of the both the cellular and humoral arms [4]. Despite being considered mainly a macrophage and T-cell driven disease, B-cell hyper-activation seems to have a role: polyclonal hyperglobulinemia has been historically associated to pulmonary sarcoidosis and more recently, different reports have documented serum positivity for antibodies directed towards nuclear antigens (ANA) and other intracellular proteins [5–8]. Their clinical significance and presence, however, is still a matter of debate.

Although the histological hallmark of the disease being the intraparenchymal formation of non-caseating granulomata [9], a disease-related vascular involvement has been described in many reports: macrovascular damage has been documented both in terms of accelerated atherosclerosis, highlighted by impaired lipid profiles and ultrasound-detected atherosclerotic lesions, and, more rarely, by vasculitic lesions potentially affecting vessels of any caliber with clinical pictures mimicking microscopic polyangiitis or Takayasu's arteritis [10].

Instead, sarcoidosis-associated microvascular damage is supported by histological data and serological markers of endothelial damage encompassing this component as part of the disease pathophysiology [11–13]. The occasional overlap between sarcoidosis and Raynaud’s phenomenon (RP) suggests microvascular implications embedded in the disease, but the dimension of the involvement has not been thoroughly investigated [14].

In this respect, nailfold videocapillaroscopy (NVC) represents a non-invasive instrumental tool mostly used to study the microvascular status in connective tissue diseases (CTDs) helping to differentiate a primary from a secondary RP as a possible very-early microvascular morphological marker of an underlying systemic disease [15–17].

This cross-sectional study aimed to describe NVC findings in sarcoidosis and report the prevalence of serum positivity for anti-nuclear (ANA) and extractable nuclear antigen (ENA) autoantibodies in sarcoidosis patients, comparing them with age- and sex- matched healthy controls (HCs) and with patients with primary Raynaud’s phenomenon (PRP). Secondarily, we investigated potential correlations between microvascular status/damage, detected by NVC, with the occurrence of autoantibodies and clinical phenotype of the disease.

## Material and methods

### Study population

A cross-sectional retrospective study was conducted in accordance with the principles of the Declaration of Helsinki and Good Clinical Practice. Twenty-seven (27) sarcoidosis patients were retrospectively selected from the recent folders of the Microvascular Unit of the Rheumatology Division of Genova University, as referral Center for the Pneumological Unit of the Hospital. All patients undergoing NVC are routinely asked to provide a written informed consent for the retrospective utilization of their anonymized images for research purposes (CONSAZHQA_0001).

Similarly, 30 PRP patients and thirty 30 HCs, age and sex matched, were selected from the large database of the Microvascular Unit founded since 1984. More specifically, we selected middle-aged adequate control individuals as PRP patients who were assessed in the past 6 months by our Microvascular Unit excluding under aged people (< 18 years old) or patients > 80 years old. Instead, HC were mainly recruited among health professionals working in our Polyclinic willing to participate to the study (i.e., nurses, administrative staff, and physicians).

Therefore, NVC figures and their detailed analysis and scores, for sarcoidosis and PRP patients, as well as for HCs, were retrospectively obtained by extracting the files from the central NVC database and considering their baseline performance. The complete medical history and laboratory findings of all enrolled patients were achieved from patient files.

The diagnosis of sarcoidosis was made according to the 2020 guidelines of the American thoracic society [18].

The exclusion criteria for sarcoidosis patients comprehended the following: patients under age (< 18 years old), active smokers, underlying malignancies, systemic untreated infections (i.e. HBV, HCV and HIV) and comorbidities, which could represent a bias for microvascular assessment at NVC, such as overlapping connective tissue diseases (systemic sclerosis (SSc), inflammatory idiopathic myopathies (IIM), systemic lupus erythematosus (SLE), Sjogren’s syndrome, Rheumatoid Arthritis (RA), mixed connective tissue disease (MCTD) and undifferentiated connective tissue disease (UCTD)), diabetes, severe uncontrolled systemic hypertension and peripheral atherosclerotic diseases. Other overlapping autoimmune diseases which could have biased the results of the immunological profile (ANA and ENA), such as Hashimoto’s thyroiditis and primary biliary cholangitis, were excluded.

PRP patients presented symmetrical and biphasic attacks, absence of signs of necrosis, negative ANA test, (titre ≤ 1:40), normal capillaroscopy and no history of CTDs [19].

### NVC examination

NVC was performed in sarcoidosis, PRP patients and HCs by the same physician (CP) blinded to the patient’s clinical history, using a 200 × magnification optical probe connected to an image analysis software (DS Medica Srl Videocap ©, Ver 10.00.13, Milan, Italy).

As for standard protocol each patient waited for a minimum of 15 min in a room at a temperature range of 20–22 °C before NVC. Two digital pictures of two-millimeter area in the middle of the nailfold bed of eight fingers, thumbs excluded, were collected for each subject [20].

According to the most recent data, the following capillaroscopic parameters have been assessed: capillary dilations (irregular or homogeneous increase of capillary diameter between 20 and 50 μm; capillaries with a diameter < 20 μm were defined as normal), giant capillaries (homogeneously dilated normal shaped loops with a diameter ≥ 50 μm), microhemorrhages (due to hemosiderin deposit), abnormal shapes (branched “bushy” capillaries, sign of neoangiogenesis) and capillary number per linear mm (abnormal if capillary density < 7 capillaries/mm) [20]. The validated semiquantitative rating scale by Cutolo et al. has been adopted to score each of the five NVC parameters (0, no changes; 1, < 33% of capillary alterations/reduction; 2, 33–66% of capillary alterations/reduction; 3, > 66% of capillary alterations/reduction per linear millimeter) [21].

In addition, the mean absolute capillaries count per linear millimeter (capillary density), has been calculated with the same standardized methodology, considering all the 16 images collected for each subject [22].

The “scleroderma pattern”, if present, was assigned according to the 2019 Fast Track algorithm by Smith et al. [23].

### Detection of autoantibodies

All the three cohorts of patients, at the baseline underwent to blood tests for ANA and extractable nuclear antigen autoantibodies (ENA). ANA were evaluated by indirect immunofluorescence (IIFA) on Hep-2/liver cells (EUROPLUS ANA Mosaic FA 1510–1), with a 1:80 serum dilution as a cut-off value. Instead, ENA had been tested through ELISA (EUROASSAY Anti-ENA ProfilePlus 1 ELISA IgG, EA 1590-1G). The kit ENA-Abs ELISA uses purified antigens of HEp-2 cell nuclei and nucleoli extract, spiked with highly purified antigens, Sm, RNP/Sm, Ro/SSA, La/SSB, Scl70 and Jo1. An enlarged panel was further prescribed to patients in case of a clinical suspicion of an overlapping scleroderma-spectrum disorder. In these subtypes of patients, antibodies against Scl-70, centromere A and centromere B, RNA polymerase III (RNAP-III), specifically RNAP-III 11 kDa (RP11) and RNAP-III 155 kDa (RP155), Ro-52, PM-Scl75, PM-Scl100, Ku, fibrillarin, Nor90, Th/To and Platelet-derived growth factor receptors (PDGF-R) were assessed. Tests were performed according to the manufacturer’s instructions (EUROIMMUNAG, Lu beck, Germany).

### Assessment of patients with sarcoidosis

All sarcoidosis patients underwent clinical evaluation, laboratory and instrumental exams as part of their routinary follow-up at their Pneumological Unit within at least 6–8 months before NVC examination, with a focused assessment on the pulmonary and extra-pulmonary involvement and severity.

Pharmacological therapy for all patients was unchanged within 3 months before NVC evaluation. The presence of extra-thoracic involvement was defined according to the 2014 World Association of Sarcoidosis and Other Granulomatous Diseases (WASOG) organ assessment instrument, including only the clinical scenarios classified as highly probable or at least probable [24].

### Laboratory investigations

Among the routine laboratory parameters of sarcoidosis patients, we performed the statistical analysis for white blood cells (WBC), haemoglobin (Hb), platelets (PLT), C-reactive protein (CRP), 25-hydroxy-vitamin D (25OH-D), serum calcium (Ca) and angiotensin converting enzyme (ACE) concentrations, the latter two being considered, among the routinary serum biomarkers, the ones most correlating with sarcoidosis disease activity [25]. Blood samples were collected, at most, 3 months before NVC examination. ACE activity was determined with a fluorometric assay (reference range of normality of 8–52 U/L).

### 18F-fluorodeoxyglucose (FDG) positron emission tomography (FDG PET)/Computed Tomography (CT)

Patients with sarcoidosis performed a total body 18-FDG-PET/CT, used to evaluate the inflammatory burden in the thoracic area (lungs and mediastinal lymph nodes) and the possible extra-pulmonary organ involvement. PET raw data were merged with the whole CT dataset by an integrating software (Syngo Image Fusion; Siemens Erlangen, Germany) which created anatomical images overlapped with FDG uptake. All PET scans were interpreted by the same nuclear medicine physician, blinded to the patient’s clinical history. FDG uptake was spatially identified by drawing a region of interest (ROI) around the pathological area of the trans-axial slice obtained by CT. For whole-body evaluation, we extrapolated the maximum standard uptake value (SUVmax), a quantitative variable measuring FDG hyper-uptake, calculated as the maximal pixel activity within the ROI. The results of PET scans in the lungs, lymph nodes, or extra-thoracic tissues were scored as “positive” if a cut-off threshold of SUVmax of 2.5 was reached or exceeded [26].

### Pulmonary function tests (PFTs)

Sarcoidosis patients underwent PFTs (including plethysmography) to measure percent predicted forced vital capacity (FVC%), forced expiratory volume in 1 s (FEV1%), diffusing capacity of carbon monoxide (DLCO%) and total lung capacity (TLC%).

### Radiological evaluation: Scadding staging system (SSS)

Sarcoidosis patients were classified according to the SSS classification (based on thoracic X-Ray) in the following five radiological sarcoidosis stages: stage 0 (normal), stage I (Bilateral hilar lymphadenopathy (BHL)), stage II (BHL plus pulmonary involvement), stage III (pulmonary involvement without BHL) and stage IV (extensive fibrosis with distortion or bullae) [27].

### Statistical analysis

Continuous variables were reported as mean value and standard deviation (SD) or median and interquartile range (IQR) when appropriate, with categorical variables as count and percentage. Chi squared test or Kruskal–Wallis rank sum test was used to explore the heterogeneity of the characteristics by subject group. Fisher's exact test or Mann–Whitney test adjusted with Bonferroni method for multiple comparisons was used for head-to-head comparisons. Spearman's rank correlation was used to calculate the relationship between ordinal variables. whereas Pearson's correlation analysis was used for metrically scaled variables.

A post-hoc power analysis was performed using G*Power version 3.1.9.7 for the estimation of the statistical power of this study: the effect size d was determined after imputing the means and standard deviations of the capillary count of patients with sarcoidosis vs healthy controls (primary endpoint) setting a significance criterion of α = 0.05. The results of power analysis are reported in Supplementary Fig. 1.

Any *p* values equal or lower than 0.05 were considered statistically significant.

Datatab® Statistics Calculator and G*Power version 3.1.9.7 were used for the statistical analysis.

## Results

### Demographic, NVC findings and autoantibody profile and in SPs, PRP patients, HCs

Among the 27 sarcoidosis patients, one patient was excluded for an overlapping systemic sclerosis with an autoantibody profile positive for anti-Scl70 and a “scleroderma pattern” detected at NVC. The remaining 26 patients showed a median age of 56.5 ± 12.5 years and 53.8% of females.

Demographic data, auto-antibody profile and capillaroscopic findings of the three cohorts are summarized in Table 1.

**Table 1:** Comparison of demographic features, NVC finding and the presence of autoantibodies between sarcoidosis patients, primary Raynaud’s phenomenon patients and healthy controls

	Sar, *N* = 26	PRPs, *N* = 30	HCS, *N* = 30	*p*-value, [95% CI]		
				Sar vs PRPs	Sar vs HCs	PRPs vs HCs
Sex female, *N* (%)	14 (53.8)	17 (56.7)	17 (56.7)	0.71	0.39	0.78
Age, mean ± SD (median, [IQR])	56.6 ± 12.6, (54.8, [46.2–62.7])	54.3 ± 14.7 (54, [42.5–67.75])	56.3 ± 10.9 (55, [48.5–64.9])	0.907, [ – 7.56, 8.49]	0.681, [ – 8.68, 5.71]	0.56, [ – 8.64, 4.73]
Body mass index, mean ± SD (median, [IQR])	26.2 ± 4.17 (25.8, [22.8–27.8])	25 ± 4.4 (24.7, [22.3–27.1])	25.5 ± 4.7 (25.1, [22.4–27.6])	0.291, [ – 1.08, 3.53]	0.539, [ – 1.63, 3.09]	0.667, [ – 2.81, 1.81]
Ex-smokers, *N* (%)	11 (42.3)	8 (26.7)	10 (33.3)	0.22	0.49	0.57
Treatment with GC, *N* (%)	21 (80.7)	0 (0)	0 (0)	** < 0.000001**	** < 0.000001**	∼1
Treatment with immunosuppressants, *N* (%)	9 (34.6)	0 (0)	0 (0)	** < 0.000001**	** < 0.000001**	∼1
Treatment with anti-platelet drugs, *N* (%)	4 (15.4)	2 (6.7)	1 (3.3)	0.29	0.11	0.55
Treatment with anticoagulants, *N* (%)	4 (15.4)	1 (3.3)	2 (6.7)	0.11	0.29	0.55
*Auto-antibodies, N positive patients* (%)						
ANA	11 (42.3)	0 (0.0)	4 (13.3)	**0.001**	**0.015**	0.362
ENA	1 (3.8)	0 (0.0)	0 (0.0)	∼1	∼1	∼1
*NVC findings*						
Dilations *N* (%)	25 (96.2)	22 (73,0)	13 (43.3)	0.34	** < 0.001**	**0.02**
Absence (0%)	1 (3.8)	8 (26.7)	17 (56.7)			
> 0%, < 33%	22 (84.6)	17 (56.7)	11 (36.7)			
> 33%, < 66%	3 (11.5)	5 (16.7)	2 (6.7)			
Microhaemorrhages, *N* (%)	9 (34.6)	6 (20.0)	5 (16.7)	0.119	0.062	0.74
Absence (0%)	17 (65.4)	24 (80.0)	25 (83.3)	0.248 (*p* for trend = **0.046**)		
> 0%, < 33%	9 (16.7)	6 (20.0)	5 (16.7)			
Neoangiogenesis, *N* (%)	10 (38.5)	0 (0.0)	1 (3,3)	**0.001**	**0.003**	0.99
Absence (0%)	1 (61.5)	30 (100.0)	29 (96.7)			
> 0%, < 33%	9 (34.6)	0 (0.0)	1 (3.3)			
> 33%, < 66%	1(3.8)	0 (0.0)	0 (0.0)			
Capillary number reduction, *N* patients (%)	3 (11.5)	1 (3.3)	0 (0.0)	0.15	**0.03**	0.31
Absence (0%)	23 (88.5)	29 (96.7)	30 (100.0)			
> 0%, < 33%	3 (11.5)	1 (3.3)	0 (0.0)			
Absolute capillary number, mean ± SD (median, [IQR])	8.26 ± 1.16 (8.4, [7.6–9])	9.45 ± 1.13 (10, [9, 10])	10.57 ± 1.01 (10.5, [10, 11])	**0.001, [** – **1.96, ** – **0.62]**	** < 0.001, [** – **3.05, ** – **1.76]**	**0.002, [** – **1.67, ** – **0.56]**

Values in bold are statistically significant (*p* < 0.05)

*ANA* anti-nuclear antibodies, *ENA* extractable nuclear antigen, *HC(s)* healthy control(s), *n* number, *NVC* nailfold videocapillaroscopy, *PRPs* primary Raynaud’s phenomenon patients, *SD* standard deviation, *Sar* sarcoidosis patients

Considering the capillaroscopic findings in sarcoidosis patients, 25 (96.2%) presented capillary dilations, 9 (34.6%) microhaemorrhages, 10 (38.5%) abnormal shapes and 3 (11.5%) a capillary number reduction. The mean absolute capillary count was 8.26 (± 1.16) capillaries/mm. Giant capillaries were not reported in any group.

**Fig. 1:**
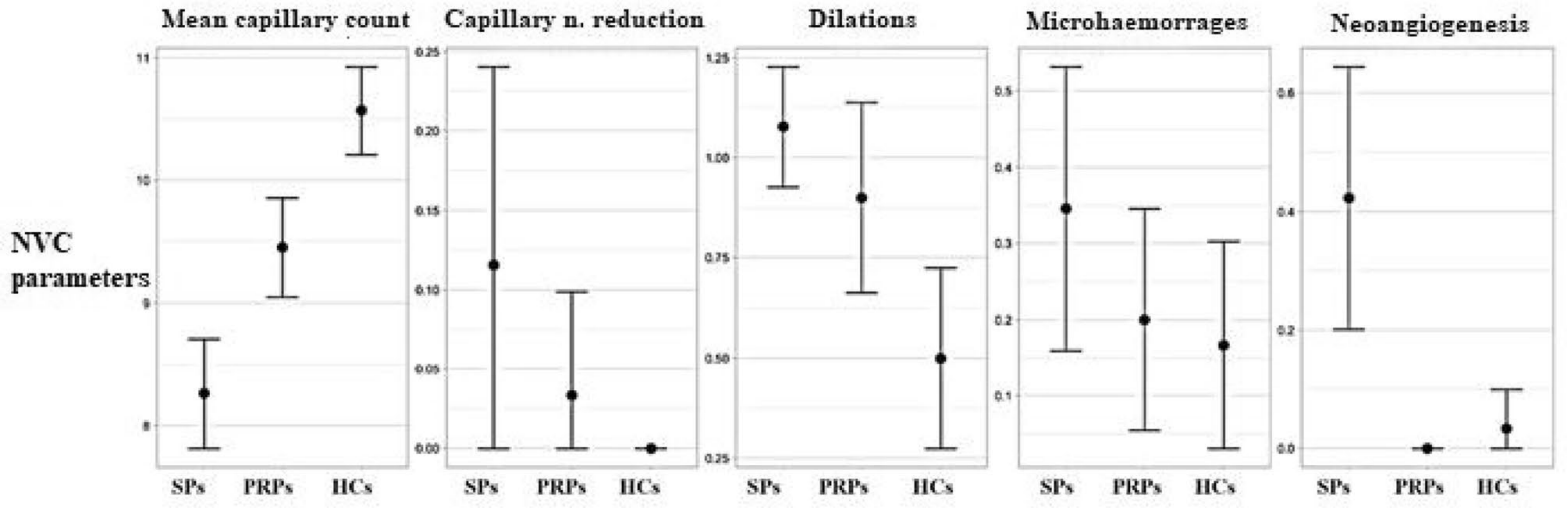
Nailfold videocapillaroscopy (NVC) findings’ comparison between sarcoidosis patients (SPs), primary Raynaud’s phenomenon patients (PRPs) and healthy controls (HCs). *n*. number

Sarcoidosis patients displayed a significantly higher rate of capillary dilations than HCs (*p* < 0.001) and a higher percentage of abnormal shapes than both PRP patients (*p* < 0.001) and HCs (*p* = 0.003). (Table 1 and Fig. 1).

When microhaemorrhage frequency was analysed, no statistically significant difference was detected through a direct comparison of the three subgroups, although we found a trend of increase in the percentage of microhaemorrhages in sarcoidosis patients higher than PRP patient who, in turn, showed a higher rate than HCs (*p* for trend = 0.046).

Additionally, sarcoidosis patients showed a mean lower capillary number for mm in comparison with both PRPs and HCs (*p* < 0.001).

When ANA and ENA positivity was assessed in the 3 subgroups, 11 sarcoidosis patients were ANA positive (42.3%, 4 patients displaying a 1:80 titre, four a 1:160 titre and three a 1:320 titre) with only one patient showing ENA (3.8%) positivity (in particular, Ro52); only 4 HCs presented ANA positivity (13.3%), but no ENA. Instead, PRP patients were, for definition, all negative for ANA and ENA tests. In particular, the prevalence of ANA positivity in sarcoidosis patients was significantly higher in comparison with PRP patients (*p* = 0.001) and HCs (*p* = 0.015).

### Assessment of sarcoidosis patients: descriptive analysis and correlations between NVC findings with functional, imaging and laboratory parameters

The clinical, laboratory, instrumental and treatment data of sarcoidosis patients are reported in Table 2. All patients had a histological-based diagnosis. The median of the disease duration was 28.4 months (± 55) with a mean onset age of 52.8 (± 4.4) years.

**Table 2:** Descriptive analysis of the cohort of sarcoidosis patients

Sex male, *n* (%)	14 (53.8)
Age, mean (SD) years	56.6 (12.6)
Weight, mean (SD) Kg	75.3 (14.7)
Height, mean (SD) cm	169.1 (9.1)
Disease Duration, median [IQR] months	28.4 (7.9, 57,7)
Histological diagnosis, *n* (%)	26 (100)
Age at disease onset, mean (SD) years	52.8 (4.4)
Former smokers, *n* (%)	11 (42.3)
Scadding radiological stage, *n* (%)	
Stage 0	3 (11.5)
Stage 1	12 (46.2)
Stage 2	8 (30.8)
Stage 3	3 (11.5)
Stage 4	0 (0)
Suv_max_, mean (SD), abs	4.4 (4.2)
Extrathoracic involvement, *n* (%)	15 (57.7)
WBC, mean (SD) * 10^9^/L	7.7 (2.8)
Hb, mean (SD) g/dL	13.2 (1.8)
PLT, mean (SD) * 10^9^/L	260.4 (78.9)
CRP, mean (SD), mg/L	17.3 (25.5)
25-OH D, mean (SD), ng/mL	22.5 (8.6)
Calcium, mean (SD), mg/dL	9.4 (0.5)
ACE, mean (SD) U/L	44.3 (28.6)
Raynaud Phenomenon, *n* (%)	3 (11.5)
ANA, *n* (%)	11 (42.3)
ENA, *n* (%)	1 (3.8)
Therapy, *n* (%)	
Naïve to therapy	
Therapy with GCs	7 (26.9)
Ongoing	8 (30.8)
Previous	19 (73.1)
Therapy with cs-/b- DMARDs	8 (30.8)
Pulmonary function tests, mean (SD) %	
FVC %	95.8 (14.7)
FEV1%	87.8 (17.4)
DLCO %	82.2 (14.4)
TLC %	91.4 (12.9)

*ACE* angiotensin converting enzyme, *ANA* anti-nuclear antibodies, *DLCO%* percent predicted diffusing lung capacity of carbon monoxide (CO), *DMARDs* Disease Modifying Anti-Rheumatic Drugs, *ENA* autoantibodies towards extractable nuclear antigens, *FEV1%* percent predicted forced expiratory volume in 1 s, *FVC%* percent predicted forced vital capacity, *GCs* glucocorticoids, *IQR* interquartile range, *n* number, *SD* standard deviation, *SUV*_*max*_ maximum standard uptake volume, *TLC%* percent predicted total lung capacity, *WBC* white blood cells, *Hb* haemoglobin, *PLT* platelets, *CRP* C-reactive protein, *25-OH D* 25-hydroxyvitamin D, *ACE* angiotensin converting enzyme

Among patients with sarcoidosis, 15 showed an extra-thoracic involvement (57.7%) whereas 3 (11.5%) did not display a pulmonary involvement at chest X-ray (SSS stage 0). Between patients with pulmonary involvement, 12 (46.2%) presented pulmonary involvement ar stage 1, 8 (30.8%) at stage 2, 3 (11.5%) at stage 3 whereas no one was at stage 4 (0%). At the FDG PET evaluation the mean SUVmax was 4.4 (± 4.2), and 8 patients (30.8%) were considered PET- negative.

Concerning current and previous treatment, 19 (73.1%) of patients received, at least once, systemic glucocorticoids (GCs) during the course of the disease and 8 (30,8%) of them were assuming GCs at the time of NVC. Additionally, 8 patients (30,8%) had an history of treatment with conventional synthetic or biologic disease modifying anti-rheumatic drugs (DMARDS); 7 (26.9%) were naïve to therapy.

**Fig. 2:**
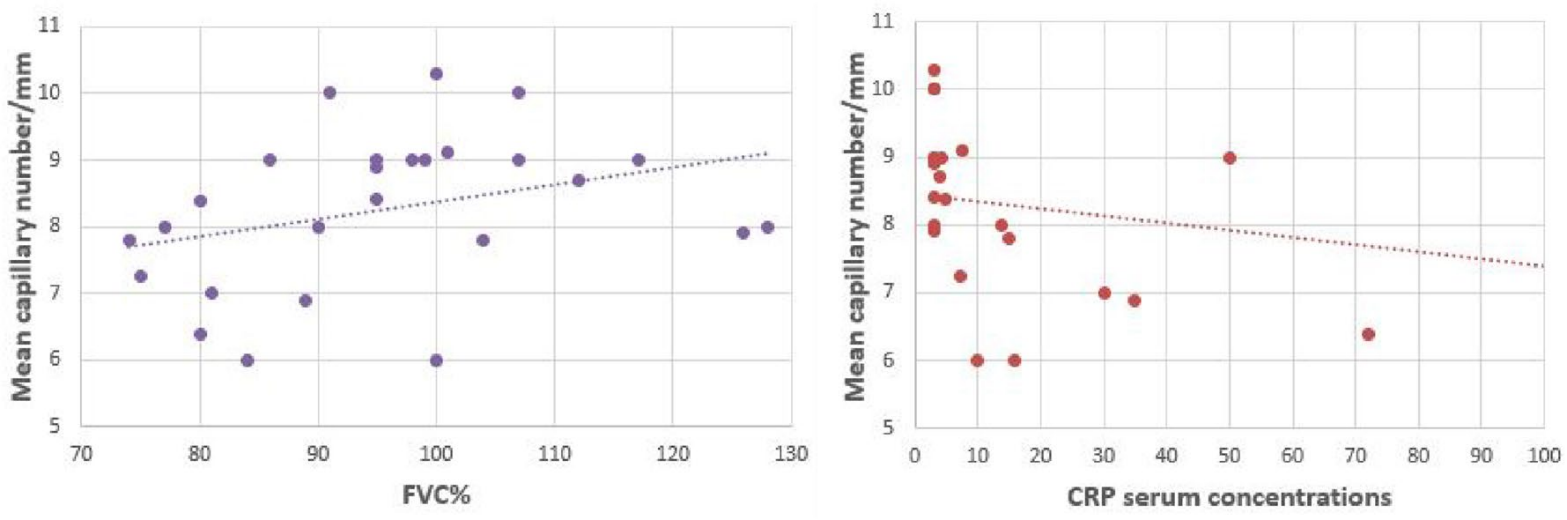
Correlations between mean capillary number and FVC% and CRP serum concentrations. *FVC*% forced vital capacity percentage, *CRP* C-reactive protein

When NVC variables were correlated with other features, negative correlations were detected between serum ACE concentrations with the presence of capillary dilations (rho =  – 0.45, *p* = 0.04) and between CRP serum concentrations and the mean capillary number (rho =  – 0.49, *p* = 0.02). Instead, a positive significant correlation of moderate strength between mean absolute capillary count and the FVC% (rho = 0.40, p = 0.04) was detected (Fig. 2).

Additionally, when sarcoidosis patients were subdivided into ANA + and ANA- patients, a significant difference emerged in the mean SUVmax, higher in the ANA + subgroup (6.27 vs 2.69, *p* = 0.01). This finding was examined on multivariate analysis, by including, as independent variables, both previous treatment with GCs and DMARDs and ANA positivity on predicting SUVmax: nevertheless, only ANA positivity justified the differences as an individual predictor (*p* = 0.01 for ANA positivity and, respectively, *p* = 0.4 and *p* = 0.86 when previous treatment with GC and DMARDs were considered). No other differences emerged in the other laboratory, imaging, and functional variables between ANA + and ANA- sarcoidosis patients (see Supplementary Table 1).

At last, no significant association was detected between NVC parameters of sarcoidosis patients with disease location, duration, PFTs, SSS, PET/CT data and ANA/ENA positivity (Table 3), and ANA/ENA positivity. NVC parameters did not significantly change also when patients were stratified according to previous treatment.

**Table 3:** Correlation analysis between nailfold videocapillaroscopy findings and sarcoidosis patients’ clinical and instrumental features

	ρ_s_ (*P*- value)		
	Mean absolute capillary count	Dilations	Neoangiogenesis
ACE%	0.20 (0.325)	– **0.40 (0.045)**	– 0.02 (0.911)
FEV1%	0.34 (0.367)	0.13 (0.444)	– 0.13 (0.792)
FVC%	**0.40 (0.041)**	0.18 (0.39)	– 0.36 (0.061)
TLC%	0.28 (0.185)	– 0.05 (0.807)	0.08 (0.707)
DLCO%	0.45 (0.068)	0.36 (0.081)	– 0.01 (0.946)
Disease duration	– 0.17 (0.402)	– 0.25 (0.221)	0.35 (0.077)
Scadding Staging System	0.01 (0.974)	– 0.11 (0.593)	– 0.06 (0.77)
SUV_max_	– 0.11 (0.583)	0.02 (0.91)	– 0.08 (0.712)

Values in bold are statistically significant (*p* < 0.05)

*ρ*_*s*_ Spearman’s rho, *ACE* angiotensin converting enzyme, *DLCO%* percent predicted diffusing lung capacity of carbon monoxide (CO), *FEV1*% percent predicted forced expiratory volume in 1 s, *FVC*% percent predicted forced vital capacity, *SUV*_*ma*x_ maximum standard uptake volume, *TLC*% percent predicted total lung capacity

## Discussion

Our results suggest that microvascular features in sarcoidosis, evaluated through NVC, might present a higher rate of nonspecific abnormalities when compared with HCs and with PRP patients.

In the present study, none of the patients showed NVC-specific abnormalities (such us giant capillaries or a capillary count < 3 capillaries/mm) mostly typical of some CTDs (i.e., SSc), ascribing all of them in the non-scleroderma pattern category according to the 2019 Fast Track algorithm by Smith et al. [23].

On the other hand, the detailed results here observed from the NVC analysis of the microvascular array in sarcoidosis patients, namely the higher prevalence of capillary dilations, the lower mean absolute capillary count and higher rate of neoangiogenesis versus adequate controls, seems to support a potential microvascular damage in sarcoidosis as recently reported [11, 13].

Indeed, the activation of the angiogenetic pathway and the presence of an impairment between angiogenesis and angiostasis involving vascular endothelial growth factor (VEGF) signalling have been already described in sarcoidosis [28–30].These findings might corroborate the angiogenetic impairment as a pathophysiological aspect of the disease and as reactive to the capillary number reduction. Such condition has been described in patients affected by SSc, where chronic increased circulating levels of VEGF are related to capillary dilations, capillary loss and reactive neoangiogenesis to tissue ischemia [31].

We observed a mild increase in trend rate of haemorrhages in sarcoidosis patients compared with HCs and PRPs. The increase in microhaemorrhages in some CTDs, such as SSc or IIM, characterized by a scleroderma or “scleroderma-like” NVC pattern, but also in other conditions such as SLE, has been documented in recent papers [32, 33]. Peculiar “comb-like” microhaemorrhages have been reported at NVC in patients affected by antiphospholipid syndrome [34].

**Fig. 3:**
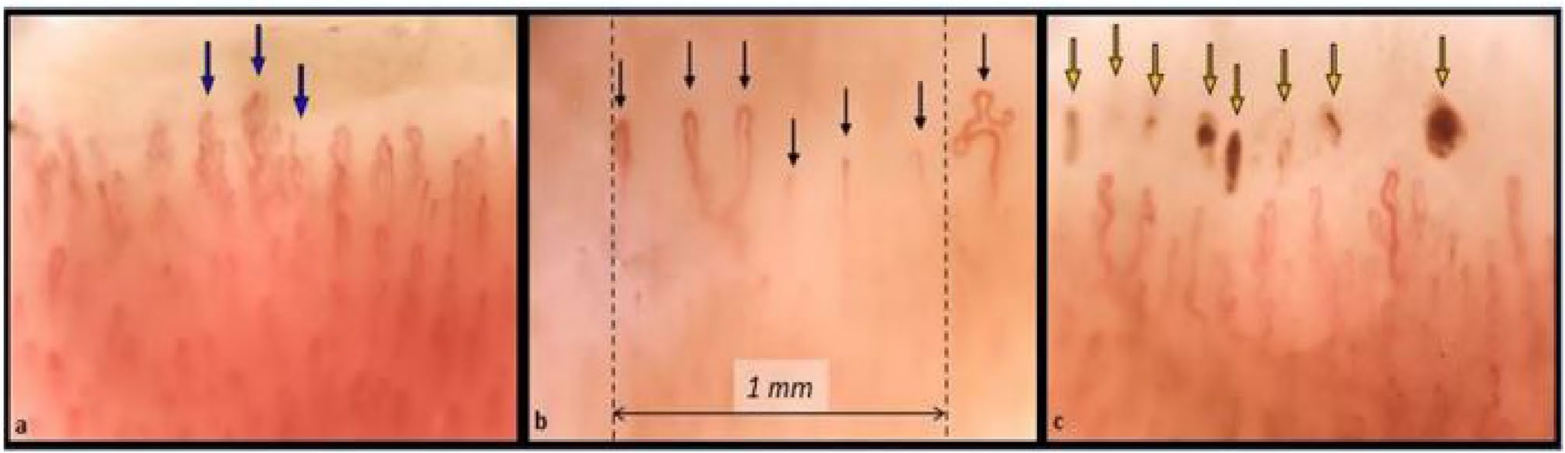
Examples of three nailfold videocapillaroscopy images recorded among three different sarcoidosis patients. (Magnification 200x). **a** Blue arrows indicate the presence of “bushy” capillaries, sign of neoangiogenesis. **b** Black arrows indicate the capillaries of the first row. The capillary count per linear mm results in six capillaries/mm (slightly reduced). **c** Yellow arrows show the presence of hemosiderin deposits due to microhaemorrhages

The implication of isolated microhaemorrhages is not clear; although being a non-specific NVC feature, they could potentially reflect a certain degree of endothelial damage. In the present study a significant amount of NVC microhaemorrhages was observed within sarcoidosis patients (34.6%). This increase could be related to the endothelial damage already reported in sarcoidosis and/or to a VEGF-induced increase in vascular permeability. On the other hand, in some sarcoidosis patients (4/26), microhaemorrhages could also be hypothetically linked to the use of anti-platelet therapy. (Fig. 3).

The prevalence of Raynaud’s phenomenon in our cohort, estimated at 11.5% (3/26 patients), is in line with epidemiological data reporting the overlapping of RP with sarcoidosis [14].

Despite being an uncommon sign, the presence of RP in patients with sarcoidosis not only highlights a likely microvascular impairment in the pathophysiology of the disease but might also indicate a subset of sarcoidotic patients characterized by microvascular detectable abnormalities worthy of a longitudinal follow-up.

Of note, all sarcoidosis patients presenting RP (3/26), showed ANA positivity without meeting any classification criteria for CTDs. Instead, the prevalence of ANA positivity in our cohort of sarcoidosis patients (42.3%) is in line with other published data detecting a positivity in up to 28%–30% of sarcoidosis patients [6, 8].

This finding might suggest that either sarcoidosis potentially displays a common immune-mediated background with other CTDs or that the subtypes of ANA-positive sarcoidosis patients might be at higher risk of developing a future overlapping CTD compared with ANA-negative patients [35].

In this respect, multiple case reports have described the association with sarcoidosis with SSc, Sjogren’s syndrome and SLE and besides ANA positivity, even the positivity for rheumatoid factor (RF) and anti-citrullinated peptide antibodies (ACPA) has been detected in a cohort of 41 sarcoid patients, respectively, in 22% and 8% of participants, despite the absence of rheumatoid arthritis [36].

Furthermore, the prevalence of ANA not only might be an indicator of CTD overlap but might also be an indirect hint of a background B-cell hyperactivity potentially linkable to sarcoidosis disease activity. Recent data, in fact, have reported a higher rate of complications and a lower percentage of sarcoidosis patients achieving remission among the ANA-positive subset of patients [37].

Although autoantibody production in sarcoidosis has not been related to specific target autoantigens as in other CTDs, their presence might be the epiphenomenon related to the tissue damage-derived antigenic release and consequently, mirroring an active disease [38]. This concept is suggested by findings of increased B-cell activating factor (BAFF) serum concentrations in sarcoidosis patients correlating with disease activity and with case series of refractory disease responsive to B-cell depleting therapy (Rituximab) [38, 39].

Interestingly, in our cohort of sarcoid patients, with the limitation of a low sample size between ANA + and ANA- patients, a higher SUVmax was detected in ANA + sarcoid patients suggesting either a higher disease activity among ANA + patients or a higher non-specific autoantibody production in the inflamed sites hyper-uptaking 18-FDG (i.e. lymph nodes).

The direct correlation observed between the mean absolute capillary number and FVC% generates linkage hypotheses between the worsening of pulmonary functions with the loss of capillaries detected peripherally by NVC. In this respect, the pathophysiology of lung involvement in sarcoidosis is complex and, according to recent reviews, appears to be mainly driven, in the first step, by an inflammatory pulmonary phase characterized by bilateral hilar adenopathy and, in a more advanced stage, by a diffuse fibrosis leading to a severe structural lung disease [40].

Besides the predominant lung parenchymal involvement, in a variable percentage of sarcoidosis patients (5–74%), also the granulomatous vascular damage is cause of pulmonary arterial hypertension (PAH) [41]. Therefore, an intrinsic sarcoid microvascular damage, consequential to both the vasculitic and fibrotic process, might explain the association that we detected between the reduction of both capillary density and FVC% as observed in SSc [42].

While the correlation between NVC parameters and lung involvement is well known for some CTDs, particularly in SSc, we might speculate that peripheral microvascular impairment might correlate with lung function in patients with sarcoidosis as well, albeit to a lesser extent. This concept becomes even more appealing thinking that some common cellular players such as M2 polarized macrophages, fibroblasts and molecular mediators (CCL18, TGFβ) are shared between fibrotic sarcoidosis and SSc [43–46].

Overall, our study results should be interpreted also under the light of some limitations.

First, the cross-sectional design of the study cannot intrinsically permit the drawing of cause–effect relationships between the analysed variables which should be assumed in longitudinal studies. The small sample size might also have been associated with a limited statistical power and could justify the absence of other studied correlations (i.e., NVC parameters with disease extension, current treatment, ANA/ENA positivity and radiological/PET-CT findings).

Second, instrumental exams, performed as routine follow-up did not suit very well the research purpose. In our study, we used the PET/TC to evaluate the thoracic and extra-thoracic inflammatory burden extrapolating only the SUVmax traditionally used in sarcoidosis evaluation.

However, being a non-volumetric parameter, SUVmax displays some limits in quantifying the extension of the disease activity. On the one hand, this index is useful in the evaluation of treatment response but, being so affected by the pharmacological therapy, it can make the comparison between patients quite challenging. Regarding the radiological assessment, we used the older SSS, traditionally based on chest-X-ray stages of the thoracic involvement in sarcoidosis, because of the lack of chest high-resolution computerized tomography (HRTC) comparable data. SSS correlates, however, much less with symptoms severity, extrapulmonary disease and pulmonary function tests compared with HRCT scores which have shown a better level of agreement with PFTs parameters, well describing the severity of pulmonary involvement [47].

At last, the lack of association between NVC parameters and many sarcoidosis patient features might simply reflect the complex and only partially understood aetiopathogenesis of sarcoidosis. In particular, the microvascular injury alone does not fully explain sarcoidosis-associated multisystem organ involvement and disease severity, being only a piece in a greater pathophysiological puzzle [43].

## Conclusions

To our knowledge, this is the first study conducted in a cohort of sarcoidosis patients which described the detailed peripheral microvascular status with the use of nailfold bed NVC.

Our findings suggest a higher rate of non-specific microvascular abnormalities in sarcoidosis whose detection by NVC could be useful for the screening of an overlapping connective tissue disease and for the monitoring of the phenotypes of sarcoidosis patients displaying RP.

The positivity for autoantibodies in sarcoidosis is in line with literature data suggesting, at least partially, autoimmune features of the disease or the production of autoantibodies reactive to tissue damage. Of note, the subset of ANA-positive sarcoidosis patients might be worthy of longitudinal follow-up considering the higher disease activity on PET imaging, corrected for treatment, in comparison with the ANA-negative subgroup.

## Supplementary Information

Below is the link to the electronic supplementary material.

Supplementary file1 (DOCX 79 KB)
