# Supplementary material for: Microvascular capillaroscopic abnormalities and occurrence of antinuclear autoantibodies in patients with sarcoidosis
Source: Rheumatol Int. 2022 Aug 30;42(12):2199–210. doi: 10.1007/s00296-022-05190-5 (PMC9548476; doi:10.1007/s00296-022-05190-5)
Supplement: Supplementary file 1 — Supplementary file1 (DOCX 79 KB) [file 296_2022_5190_MOESM1_ESM.docx]

| **Analyzed variable**  **(mean ± st. deviation)** | ANA+ sarcoidosis patients  n = 11 | ANA- sarcoidosis patients  n = 15 | p-value, [95% CI] |
| --- | --- | --- | --- |
| 25-OH D serum concentrations | 17.18 ± 5.46 | 25.57 ± 8.9 | p = 0.125, [-19.63, 2.83] |
| Calcium serum concentrations | 9.4 ± 0.53 | 9.38 ± 0.58 | p = 0.928, [-0.51, 0.55] |
| CRP serum concentrations | 16.92 ± 31.16 | 17.59 ± 21.59 | p = 0.952, [-23.53, 22.18] |
| WBC (x 10^9^/L) | 7.38 ± 2.78 | 8.11 ± 2.83 | p = 0.53, [-3.12, 1.65] |
| Haemoglobin (g/L) | 13.1 ± 1.96 | 13.31 ± 1.82 | p = 0.79, [-1.81, 1.39] |
| PLT (x 10^9^/L) | 240.18 ± 79.49 | 277.62 ± 77.32 | p = 0.256, [-103.97, 29.11] |
| Forced vital capacity (%) | 99.27 ± 14.74 | 93.27 ± 14.58 | p = 0.312, [-5.99, 18] |
| FEV1 (%) | 93.18 ± 18.89 | 83.93 ± 15.69 | p = 0.186, [-4.76, 23.25] |
| DLCO | 83.55 ± 14.96 | 81.2 ± 14.34 | p = 0.689, [-9.62, 14.31] |
| TLC | 94.4 ± 13.51 | 89.21 ± 12.55 | p = 0.344, [-5.94, 16.31] |
| CPI | 23.86 ± 14.2 | 28.57 ± 14.8 | p = 0.423, [-16.63, 7.22] |
| **SUVmax** | **6.27 ± 4.42** | **2.69 ± 2.68** | **p = 0.014, [0.79, 6.38]** |

**Supplementary Table 1.** Differences among ANA+ and ANA- sarcoidosis patients in the laboratory parameters, lung function tests and SUVmax

**Power analysis results**

For the t-test detecting differences between two independent means (in this case, the capillary count in patients with sarcoidosis vs healthy controls), the estimated power equalled 1.0 (> 0.8) by inputting a sample size = 26 for sarcoidosis patients and a sample size = 30 for healthy controls (Supplementary Figure 1 for the power plotted against total sample size).

**Supplementary Figure 1.** Power of t-test evaluating mean capillary number in sarcoidosis versus healthy controls plotted versus total sample size (n = 56) with an estimated power of approximately 0.905.
